# Antifungal Activity of Sesamol on *Pestalotiopsis neglecta*: Interfering with Cell Membrane and Energy Metabolism

**DOI:** 10.3390/jof10070488

**Published:** 2024-07-15

**Authors:** Weihu Ma, Jingyu Ji, Bowen Zhang, Wenzhuo Sun, Jinyan Zhao, Jie Zhang, Guocai Zhang

**Affiliations:** 1 Heilongjiang Province Key Laboratory of Forest Protection, School of Forest, Northeast Forestry University, Harbin 150040, China; 1098548982@nefu.edu.cn (W.M.); ji@nefu.edu.cn (J.J.); 18348556488@163.com (W.S.); zjyxnz@nefu.edu.cn (J.Z.); 2 School of Information and Computer Engineering, Northeast Forestry University, Harbin 150040, China; bzhang60@sina.com; 3 School of Life Sciences, Northeast Forestry University, Harbin 150040, China

**Keywords:** microscopic observation, cell membrane permeability and integrity, cell membrane integrity, virulence factor, TCA cycle, mode of action

## Abstract

This paper investigated the inhibitory effect of Sesamol (Ses) on *Pestalotiopsis neglecta*. The potential inhibitory mechanisms were explored by observing changes in cell morphology, measuring alterations in cell membrane-related indices, as well as energy metabolism-related indices and changes in enzyme activities related to virulence. The results show that Ses completely inhibited the growth of *P. neglecta* at 600 μg/mL (minimum inhibitory concentration and minimum fungicidal concentration), with an EC_50_ of 142 ± 13.22 μg/mL. As observed with scanning electron microscopy (SEM) and transmission electron microscopy (TEM), Ses treatment resulted in the breakage and crumpling of *P. neglecta* cell membrane and organelle lysis. Ergosterol content and the total lipid in *P. neglecta* treated with 300 μg/mL Ses was 91.52% and 54% of that in the control groups, respectively. In addition, spores were stained, increased leakage of intracellular constituents at 260 nm, and decreased extracellular pH. This suggests damage to the cell membrane integrity and permeability. Furthermore, Ses decreased the ATP levels and key enzymes in the tricarboxylic acid (TCA) cycle, indicating interference with the fungal energy metabolism. Moreover, the activities of polygalacturonase (PG) and endoglucanase (EG) of *P. neglecta* treated with 300 μg/mL of Ses were only 28.20% and 29.13% of that in the control groups, respectively, indicating that Ses can reduce the virulence of *P. neglecta*. In conclusion, our results show that Ses should be considered as a potential plant-derived fungicide due to its ability to disrupt the morphology of *P. neglecta*, damage cell membrane integrity and permeability in *P. neglecta*, interfere with energy metabolism, and reduce its virulence, ultimately affecting the fungal growth.

## 1. Introduction

*Pestalotiopsis neglecta* is the pathogenic fungus responsible for black spot needle blight in *Pinus sylvestris* var. *mongolia*, which also causes blueberry rot and bud blight in Japanese cryptomeria [1]. Black spot needle blight has been reported in Inner Mongolia, China, leading to significant economic losses in the region [2]. However, black spot needle blight control still heavily relies on chemical fungicides [3]. The limitations of chemical fungicides have prompted efforts to explore alternative methods for managing plant diseases.

Recently, there has been increased interest in replacing chemical biocides with natural alternatives for controlling plant pathogenic fungi [4,5,6]. Phenols are natural organic compounds synthesized by plants during normal development. They contain one or more phenolic groups and are considered secondary metabolites [7,8]. It has been proven that phenolics exhibit good antibacterial and antifungal activity [8,9]. For example, Wang et al. [10] demonstrated that eugenol exerts its antifungal effects by disrupting the plasma membrane of *Botrytis cinerea* and altering membrane permeability. In another study by Campaniello et al. [11], eugenol exhibited antifungal effects against *Penicillium*, *Aspergillus*, and *Fusarium* spp. Additionally, Mączka et al. [12] and Wang et al. [13] found that carvacrol inhibits the growth of *Alternaria solani*, *Botrytis cinerea*, and *Fusarium oxysporum*. Carvacrol and thymol can decrease the total lipid content and disrupt the cell membrane structure of *Botrytis cinerea* [14]. Kong et al. [15] showed that thymol and salicylic acid disrupted mitochondria-related functions in *Rhizopus stolonifer* by decreasing the activity of TCA cycle-related enzymes.

Sesamol (Ses) (3,4-methylenedioxyphenol) is an aromatic compound found in *Sesamum indium* oil (Pedaliaceae) (sesame oil) that exhibits various biological activities. Ansari et al. [16] demonstrated that Ses inhibited six different strains of *Candida albicans* and other clinical isolates to varying degrees. Hans et al. [17] demonstrated that Ses effectively inhibited *Mycobacterium tuberculosis* and altered its colony morphology. Determining the antifungal activity and antifungal mechanism of Ses remains challenging, especially in studies involving phytopathogenic fungi. To justify the use of Ses as an antifungal agent in plant diseases, the antifungal activity and metabolism of Ses need to be clarified.

Studies have shown that phenolics exhibit their antifungal activity by disrupting membrane structure and intracellular homeostasis, such as by damaging membrane components, disrupting membrane integrity, and affecting permeability [18,19]. In addition, dysregulation of cellular homeostasis due to phenolic treatment can impact energy metabolism and the synthesis of macromolecules as extracellular toxins [19,20,21]. Consequently, a comprehensive analysis of the impact of Ses on changes in membrane structure and metabolic metabolism will help us better understand the inhibitory mechanism in *P. neglecta*. 

This current research aimed to elucidate the inhibition mechanism of *P. neglecta* with Ses. The antifungal mechanism will be assessed by performing the following measurements: (1) the mycelial surface morphologies and microstructure of mycelial cells and the integrity of cell membrane using SEM, TEM, and inverted fluorescence microscope; (2) alterations in ergosterol and total lipid content; (3) alterations in the relative conductivity and extracellular pH; (4) the leakage of intracellular constituents at 260 nm; (5) changes in ATP content and enzyme activities within the TCA pathway; (6) changes in polygalacturonase (PG) and endoglucanase (EG) activities of *P. neglecta*.

## 2. Materials and Methods

### 2.1. Materials

Ses (99%) was acquired from Yi’en Chemical Technology Co., Ltd. (Shanghai, China), while analytical-grade reagents and solvents were procured from Fuyu Fine Chemical Co., Ltd. (Tianjin, China). The enzyme activity kit was obtained from Keming Biotechnology Co., Ltd. (Suzhou, China).

### 2.2. Pathogens and Cultures

*Pestalotiopsis neglecta* was obtained from the Key Laboratory of Forest Protection at Northeastern Forestry University in Heilongjiang Province and incubated on potato dextrose agar (PDA) at 25 °C. Spore suspensions were obtained by soaking 1-week-old mycelium in sterile water, filtering it through sterile desiccated cotton, and then adjusting it to 1 × 10^6^ CFU/mL using a hemocytometer plate.

### 2.3. Inhibition of Mycelial Growth of P. neglecta by Ses

The agar dilution method was used to investigate the inhibitory impact of Ses on *P. neglecta* [13]. Fungal mycelial plugs (Φ = 5 mm) were cultured in the center of a petri dish (Φ = 60 mm) and cultured at 25 °C for 7 d. Each petri dish contained 5 mL of PDA medium with various levels (600, 300, 150, 75, and 37.5 μg/mL) of Ses, which were dissolved in 1% dimethyl sulfoxide (DMSO). The control groups were equal amounts of sterile water and 1% DMSO solution; in addition, the positive drug was bromothalonil (600 µg/mL). The growth diameter was measured after the mycelium had spread throughout the control medium, and virulence equations, as well as minimum inhibitory concentration (MIC) and minimum fungicidal concentration (MFC), were calculated. Each assay was repeated three times. To calculate the growth inhibition rate (%), the following formula was used [22]:

$$\mathrm{Inhibition}\;\mathrm{rate}\left(\%\right)=\frac{\left(\mathrm{Growth}\;\mathrm{diameter}\;\mathrm{in}\;\mathrm{control}\;-\;\mathrm{Growth}\;\mathrm{diameter}\;\mathrm{in}\;\mathrm{treatment}\right)}{\mathrm{Growth}\;\mathrm{diameter}\;\mathrm{in}\;\mathrm{control}}\times 100$$

### 2.4. Scanning Electron Microscopy (SEM) and Transmission Electron Microscopy (TEM)

It is well known that SEM and TEM provided a powerful tool for studying the microstructure and ultrastructural lesions of pathogenic fungi [23,24]. This method combined the naked observation of mycelial growth morphology on the medium with microscopic and macroscopic analyses, making the test more credible [25,26]. Therefore, we conducted a thorough electron microscopic examination of *P. neglecta* treated with Ses.

The study investigated the effect of Ses on the micromorphology and ultrastructure of *P. neglecta* using SEM and TEM. A mycelial plug (Φ = 5 mm) was cultured on a PDA medium containing 0 and 142 μg/mL (EC_50_) of Ses. Sterile coverslips were placed at a 45° angle, 2–3 cm away from the mycelial plugs, and cultured at 25 °C for 7 d. After the mycelium growth on coverslips, mycelium samples were treated using the method outlined by He et al. [27], with slight adjustments when observed at 10,000× magnification under an SEM (EM-30 Plus, COXEM Co., Ltd., Daejeon, Republic of Korea).

The mycelial plugs (Φ = 5 mm) were placed in potato dextrose broth (PDB) at 25 °C for 3 d with a concentration of 142 μg/mL (EC_50_) Ses on a shaking incubator (HZQ-F160, Harbin Donglian Electronic Technology Development Co., Ltd., Harbin, China) set at 150 rpm. Samples without Ses treatment served as the control groups. The mycelium samples were then treated according to Xia et al. [28] and Kong et al. [15] with minor modifications. Finally, all samples were examined using TEM (JEM-2100, JEOL Co., Ltd., Tokyo, Japan) at 5000× magnification. 

### 2.5. Determination of Ergosterol Content of P. neglecta Mycelium by Ses

Three mycelial plugs (Φ = 5 mm) were placed in a triangular flask containing 190 mL of PDB medium with 10 mL of a 1% DMSO solution. The DMSO solution contained various concentrations of Ses, resulting in final concentrations of Ses (300, 150, and 75 µg/mL). The control groups were equal amounts of sterile water and 1% DMSO solution [22]. The flasks were placed in a shaking incubator (HZQ-F160, China) at 25 °C and 150 rpm for 3 d. Following incubation, the mycelium and supernatant were filtered and collected using a sterile silk cloth. Mycelial samples and supernatants were collected once every 12 h for five times. 

The protocol for measuring ergosterol content was modified based on Zeng et al. [29]. In summary, 20 mg of dried frozen mycelium powder was saponified in a mixture of 5 mL of 25% (*w*/*v*) NaOH and 65% (*v*/*v*) ethanol. The solution was mixed using a vortexer for 10 min and then placed in a water bath at 85 °C for 2 h. Next, 1 mL of sterile distilled water and 3 mL of n-heptane were added to the solution. The mixture was vigorously vortexed for 3 min and then left to cool at room temperature. The layer of n-heptane was then separated and stored at −20 °C for 24 h. Ergosterol content was examined by measuring the absorbance at 230 nm and 282 nm using a multifunctional enzyme labeler (SuPerMax 3100, Shanghai Sembcorp Bio-technology Co., Shanghai, China). The experiment was conducted three times to ensure accuracy and reliability. Finally, ergosterol (%) was based on the following formula [30]:

$$\mathrm{ergosterol}\;\mathrm{content}\;(\%)=\frac{\left(\frac{A_{282}}{290}\right)-\left(\frac{A_{230}}{518}\right)\times 100}{W}$$

Note: W is the mass of lyophilized powder of mycelium (g).

### 2.6. Determination of Total Lipid Content of P. neglecta Mycelium by Ses

The impact of Ses on the membrane composition of cells was evaluated by measuring the total lipid content using the protocols described by Elsherbiny et al. [31] and Patel et al. [32]. Mycelial lipid content was determined at various concentrations (300, 150, and 75 µg/mL). Briefly, 10 mg of mycelium powder was mixed with 1 mL of water. A methanol–chloroform mixture (1:1) was added, and the mixture was shaken for 30 min, followed by centrifugation. The 0.5 mL of chloroform substrate was mixed with 200 µL of NaCl and centrifuged again. The lower 200 µL layer was mixed with sulfuric acid and boiled for 10 min. Next, 3 mL of vanillin phosphate solution was added and left for 10 min. Finally, the absorbance at 525 nm was determined using a multifunctional enzyme labeler (SuPerMax 3100, Shanghai, China), and the total lipid content was calculated from a standard calibration curve using cholesterol as a standard. Each assay was conducted in triplicate for statistical reliability [14]. 

### 2.7. Determination of Cell Membrane Integrity

The procedure for PI staining was based on the method proposed by Li et al. [33] with slight modifications. Briefly, *P. neglecta* spore suspension (1 × 10^6^ CFU/mL) was mixed with various concentrations of Ses solution (300, 150 and 75 µg/mL) and cultured for 6 h at 25 °C with constant shaking (150 rpm). After 6 h of incubation, the precipitate was collected by centrifugation at 8500 rpm for 2 min; it was then washed thrice with phosphate-buffer saline (PBS, pH 7.2) and subsequently exposed to 1 μg/mL propidium iodide (PI). After 30 min of incubation, the precipitate was re-suspended by centrifugation at 8500 rpm for 2 min, washed thrice with PBS, and re-suspended in PBS for further observations. Finally, the sample was re-suspended in PBS for further observations. The treated samples were examined using a fluorescence inverted microscope (Axio Observer 3, Carl Zeiss Microscopy, LLC, Jene, Germany) to count the number of spore stains.

### 2.8. Determination of Cellular Content Leakage and pH Value

Refer to Section 2.4 for the procedure for collecting the supernatant. The collected supernatant (4 °C, 12,000 rpm) was centrifuged for 10 min [33,34]. The relative conductivity (μS/cm) was measured using a digital conductivity meter (DDS-11, Shanghai Yoke Instrument Co., Ltd., Shanghai, China). Each assay underwent three repetitions to ensure the robustness of the results.

In addition, the absorbance of the supernatant was evaluated at 260 nm by employing a multifunctional enzyme labeler (SuPerMax 3100, Shanghai, China), and the nucleic acid concentration was expressed as OD_260_ [35]. The supernatant was treated as described above, and the pH was measured using a pH meter (PHS-3E, Shanghai Yidian Scientific Instrument Co., Ltd., Shanghai, China) [36]. 

### 2.9. Measure of ATP Content and Key Enzyme Activities in the TCA Cycle

Spectrophotometric assays were used to measure the ATP content, as well as the activities of succinate dehydrogenase (SDH), malic dehydrogenase (MDH), citrate synthetase (CS), isocitrate dehydrogenase (IDH), and α-ketoglutarate dehydrogenase (α-KGDH) in *P. neglecta* treated with varying concentrations of Ses (300, 150, and 75 µg/mL) [37]. The ATP content and the MDH, SDH, and IDH activities were assessed using a multifunctional enzyme labeler (SuPerMax 3100, Shanghai, China) at specific wavelengths for each enzyme—340 nm for ATP content, MDH, SDH, and IDH, 600 nm for α-KGDH, and 412 nm for CS. Furthermore, enzyme activity measurements were performed in triplicate following the manufacturer’s instructions.

### 2.10. Determination of PG and EG Activities in P. neglecta 

Refer to Section 2.4 for the procedure for collecting supernatant. The collected supernatants were treated according to the manufacturer’s instructions, and the PG and EG activities were determined using a multifunctional enzyme labeler (SuPerMax 3100, Shanghai, China) [22]. Activities of PG and EG were measured at 600 nm and 412 nm, respectively. Each assay underwent three repetitions to ensure the robustness of the results.

### 2.11. Data Analysis

Data were averaged and statistically analyzed using SPSS 22.0 (IBM, Armonk, NY, USA) with one-way ANOVA (Tukey test) and Student’s T-tests to compare mean differences at a significance level of *p* < 0.05. GraphPad Prism 9 (GraphPad Software, San Diego, CA, USA) was used for graphing.

## 3. Results

### 3.1. Ses Inhibits Mycelial Growth of P. neglecta

**Figure 1:**
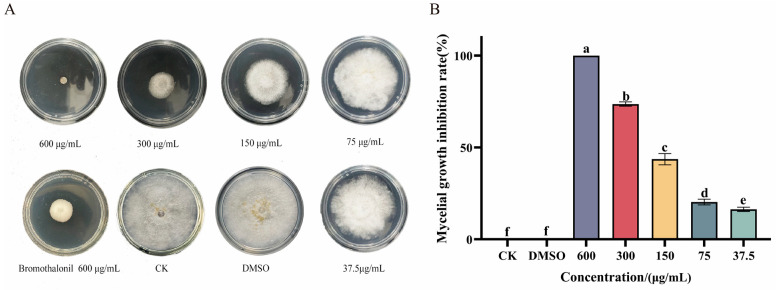
(**A**) The inhibition of mycelial growth of *P. neglecta* at 25 °C 7 days after Ses treatment; the positive drug is bromothalonil (600 µg/mL). (**B**) Inhibition of mycelial growth of *P. neglecta* at 25 °C 7 days after Ses treatment. The bars represent the standard error of the mean (*n* = 3). The different letters represent statistically significant differences between the effects of different concentrations, according to the Tukey test (*p* < 0.05).

The impact of Ses on *P. neglecta* is demonstrated in Figure 1. Ses inhibited the growth of *P. neglecta* in a concentration-dependent manner; Ses demonstrated superior inhibition to bromothalonil at the same concentration. As shown in Figure 1B, significant differences in inhibition were observed between high (600 μg/mL) and low (37.5 μg/mL) concentrations. Furthermore, 600 μg/mL of Ses resulted in the complete growth inhibition of *P. neglecta* (MIC and MFC are both 600 μg/mL). Table 1 presents the EC_50_ values and linear regression equations for Ses against *P. neglecta*.

### 3.2. Ses Destroys Membrane Integrity of P. neglecta 

**Figure 2:**
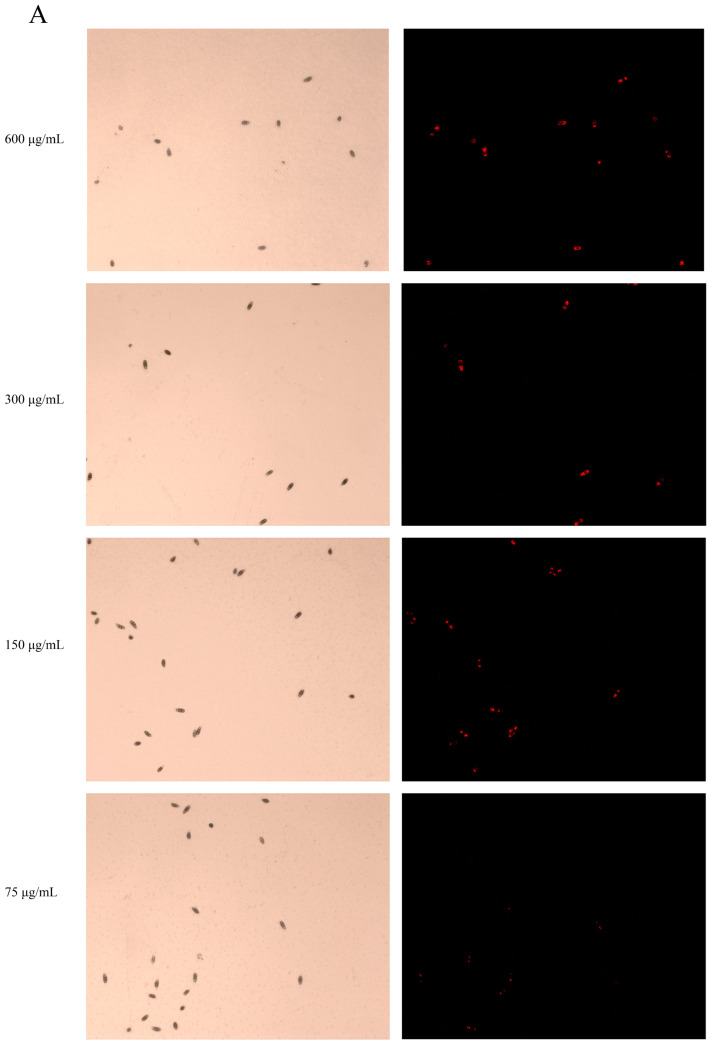

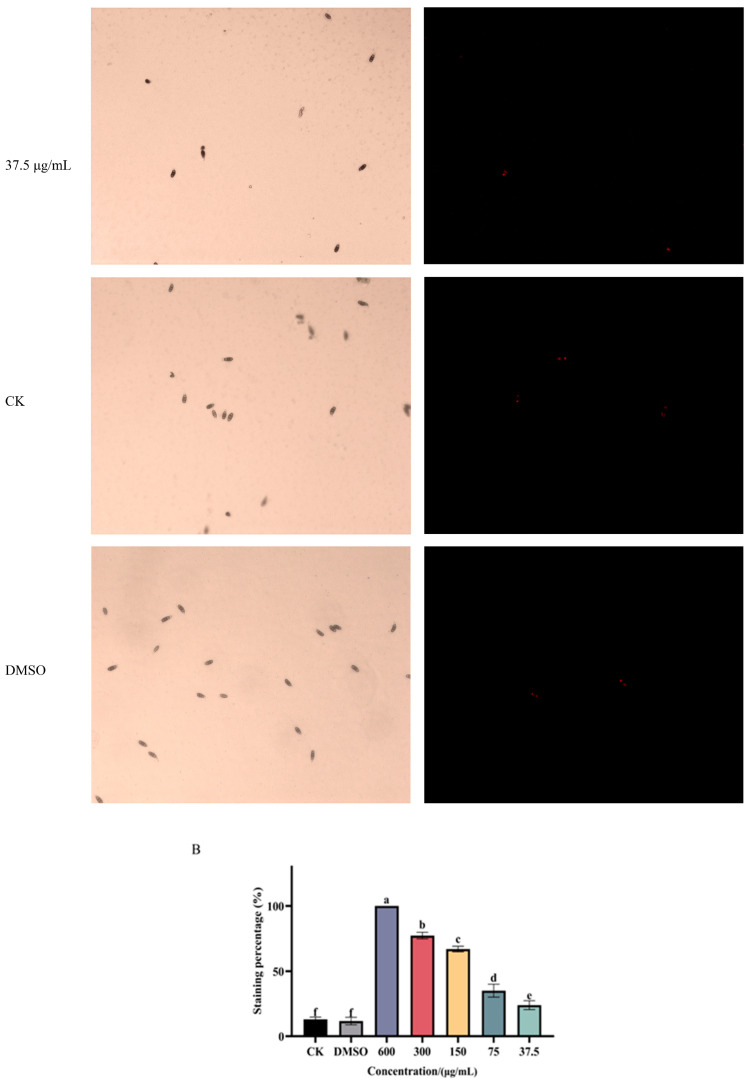
The cell membrane integrity in *P. neglecta* spores after Ses treatment. PI-stained spores of *P. neglecta* after treatment with 600, 300, 150, 75, and 37.5 µg/mL Ses for 8 h was observed at 100× (**A**). Staining percentage of spores stained after PI treatment (**B**). The different letters represent statistically significant differences between the effects of different concentrations, according to the Tukey test (*p* < 0.05).

The integrity of the cell membrane in *P. neglecta* spores after being treated with Ses was evaluated using the PI staining assay, and the results are shown in Figure 2A. The untreated spores exhibited minimal fluorescence when observed under white light and fluorescence. Conversely, spores treated with Ses displayed a distinct red fluorescence, indicating membrane damage. Figure 2B illustrates that the proportion of PI-stained spores was markedly higher in the Ses-treated groups compared with the control groups. Specifically, all spores were stained when treated with 600 µg/mL of Ses, while only 23.89% of the spores were stained at 37.5 µg/mL of Ses. These results show that Ses compromised the cell membrane integrity of *P. neglecta*.

### 3.3. Ses Alters the Morphology of P. neglecta

**Figure 3:**
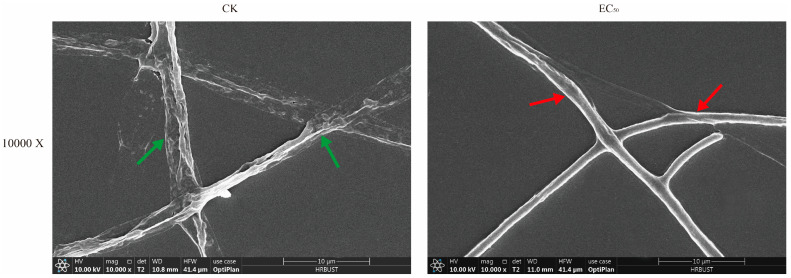
Impact of Ses on *P. neglecta* surface morphology. Red arrows represent untreated *P. neglecta* mycelium, while green arrows represent *P. neglecta* mycelium treated with EC_50_.

**Figure 4:**
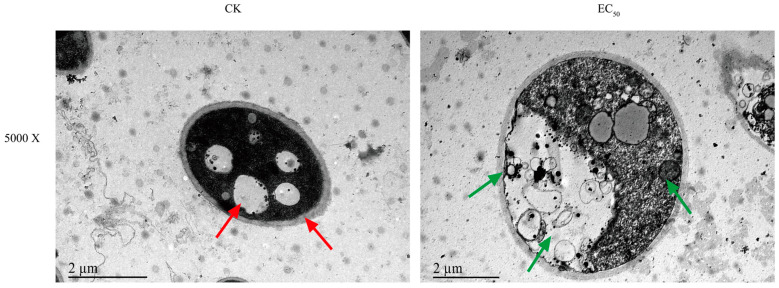
Impact of Ses on *P. neglecta* ultra-microstructure. Red arrows represent untreated *P. neglecta* cells, while green arrows represent *P. neglecta* mycelium treated with EC_50_.

Changes in mycelial surface morphology were revealed through SEM. As depicted in Figure 3, the mycelium of the control groups grew in a straight and full morphology with an intact structure, whereas the Ses-treated group at EC_50_ resulted in irregular contraction of the mycelium, morphological shrinkage, and even mycelium breakage. To further study the microstructure of mycelium, TEM was used to investigate the microstructural changes in the mycelium. The control groups of mycelium cells exhibited a visually intact cell membrane and well-preserved organelles, as illustrated in Figure 4. Conversely, the mycelium cells treated with Ses showed a wrinkled cell membrane and degraded organelles such as vesicles and mitochondria.

### 3.4. Impact of Ses on Ergosterol and Total Lipid Content in P. neglecta

**Figure 5:**
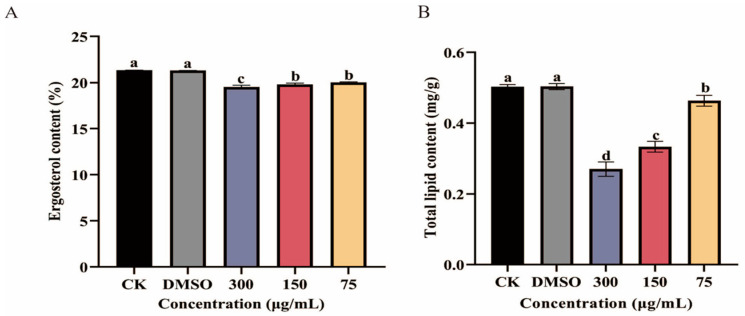
The impact of various concentrations of Ses on the total lipid content (**A**) and ergosterol content (**B**) in *P. neglecta*. The bars represent the standard error of the mean (*n* = 3). The different letters represent statistically significant differences between the effects of different concentrations, according to the Tukey test (*p* < 0.05).

The impact of various concentrations of Ses on the ergosterol content in *P. neglecta* was illustrated in Figure 5A. As shown in Figure 5A, statistically significant differences were noted between the treatment and control groups. The ergosterol content in the treatment groups of 300 µg/mL, 150 µg/mL, and 75 µg/mL was 91.52%, 92.79%, and 93.91% of that in the control groups, respectively.

As depicted in Figure 5B, the total lipid content of *P. neglecta* exhibited significant variation (*p* < 0.05) across different concentrations of Ses. The total lipid content decreased as the concentration of Ses increased. Specifically, the treatment groups of 300 µg/mL, 150 µg/mL, and 75 µg/mL exhibited reductions of 54%, 66%, and 92%, respectively, in total lipid content compared with the control groups. This demonstrates a clear negative correlation between the concentration of Ses and the total lipid content of *P. neglecta*.

### 3.5. Leakage of Cell Constituents after Ses Treatment

**Figure 6:**
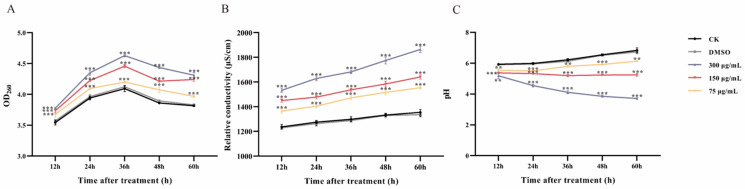
The impact of various concentrations of Ses on leakage of intracellular constituents at 260 nm (**A**), relative conductivity (**B**) and extracellular pH (**C**) of *P. neglecta*. Values were calculated as means (*n* = 3), and vertical bars represent standard errors. ** (*p* < 0. 01) *** (*p* < 0.001) vs. control group; the significant difference between DMSO and CK was ns.

The absorption peaks of nucleic acids occur at 260 nm, indicating that optical density (OD) values are directly proportional to their concentrations. Higher OD values correspond to increased concentrations of nucleic acids. The OD values of Ses-treated *P. neglecta* showed a significant increase when exposed to concentrations of 300 µg/mL, 150 µg/mL, and 75 µg/mL, as illustrated in Figure 6A, surpassing the levels observed in the control groups. After 36 h of incubation, a significant increase in the OD values of Ses-treated *P. neglecta* was observed (1.13-, 1.08-, and 1.02-fold compared with the control groups, respectively).

Relative conductivity was measured to identify the impact of Ses on the cellular leakage of *P. neglecta* mycelia (Figure 6B). In both the control and treatment groups, a dose-dependent increase in relative conductivity was observed in *P. neglecta* mycelia following treatment with Ses, indicating a significant effect. After 60 h of treatment, the relative conductivity levels of Ses at concentrations of 300 µg/mL, 150 µg/mL and 75 µg/mL were recorded to be 1.38-, 1.21-, and 1.15-fold higher, respectively, compared with control groups.

As illustrated in Figure 6C, the pH of the treatment groups exhibited a notable dose-dependent effect compared with the control groups. After 60 h of treatment, the pH in the treatment groups of 300 µg/mL, 150 µg/mL, and 75 µg/mL was 54.17%, 76.83%, and 89.75% of that of the control groups, respectively.

### 3.6. Effect of Ses on ATP Content and Key Enzyme Activities in the TCA Cycle

**Figure 7:**
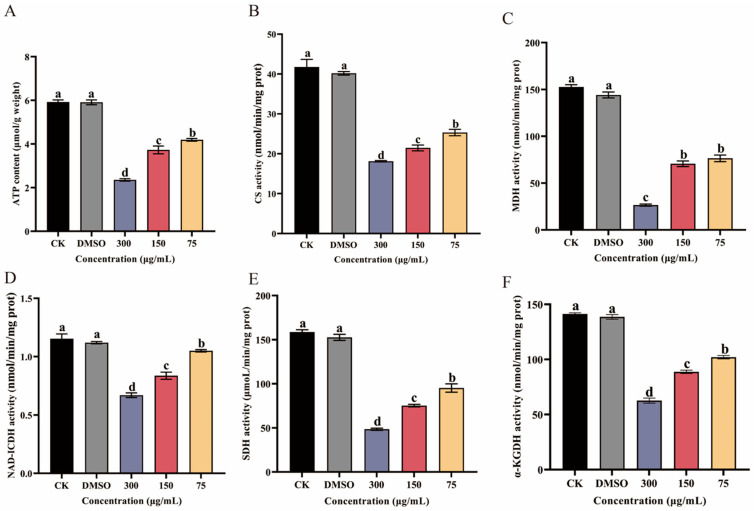
The impact of different concentrations of Ses on ATP content (**A**), CS activity (**B**), MDH activity (**C**), NAD-ICDH activity (**D**), SDH activity (**E**), α-KGDH activity (**F**) of *P. neglecta*. Values were calculated as means (*n* = 3), and vertical bars represent standard errors. The different letters represent statistically significant differences between the effects of different concentrations, according to the Tukey test (*p* < 0.05).

ATP content was measured to assess the impact of Ses on *P. neglecta* mycelial energy metabolism (Figure 7A). A marked reduction in ATP content was found following exposure to Ses at different concentrations (300 µg/mL, 150 µg/mL, and 75 µg/mL). In addition, a linear relationship between ATP content and Ses concentration was seen. At a Ses concentration of 300 µg/mL, the ATP content was only 39.8% of the control groups.

The TCA-related cycle key enzyme activities are shown in Figure 7B–F. The CS, MDH, NAD-ICDH, SDH, and α-KGDH activities in Ses-treated *P. neglecta* mycelium were markedly reduced with increasing Ses concentration compared with the control groups, and the differences were significant between the concentrations. The CS, SDH, NAD-ICDH, MDH, and α-KGDH activities of *P. neglecta* treated with 300 µg/mL Ses were 43.39%, 30.57%, 58.26%, 17.36%, and 44.35% of those in the control groups, respectively.

### 3.7. Ses Inhibits PG and EG Activities of P. neglecta

**Figure 8:**
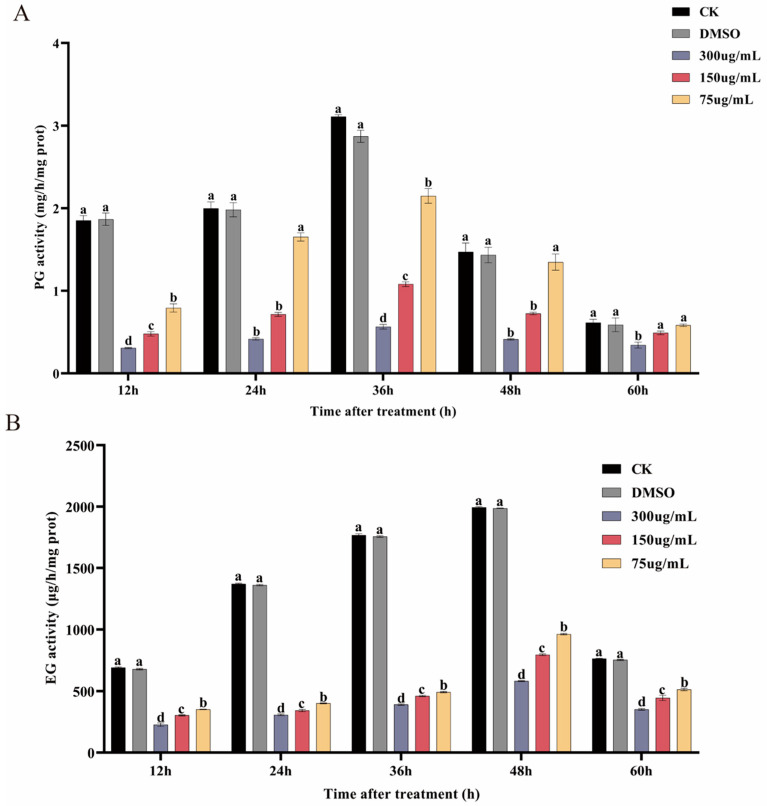
The impact of different concentrations of Ses on PG (**A**) and EG (**B**) activities of *P. neglecta*. The different letters represent statistically significant differences between the effects of different concentrations, according to the Tukey test (*p* < 0.05).

The impact of concentrations of Ses on the PG and EG activities of *P. neglecta* is shown in Figure 8. Figure 8A shows that PG activity peaked at 36 h, following an initial increase and then a decrease. There were marked differences in EG activity between the Ses-treated and control groups. At 36 h, the PG activity of *P. neglecta* treated with 300 µg/mL, 150 µg/mL, and 75 µg/mL of Ses was 28.20%, 44.44%, and 77.78% of that in the control groups, respectively. As shown in Figure 8B, the activity of EG exhibited an overall gradual decrease over time, reaching its peak at 48 h. Marked differences in EG activity were observed between the Ses-treated groups and the control groups. At 48 h, Ses concentrations of 300 µg/mL, 150 µg/mL, and 75 µg/mL resulted in EG activity of only 29.13%, 39.87%, and 48.26%, respectively, compared with those of the control groups.

## 4. Discussion

Many studies have reported that phenolic substances can strongly inhibit plant pathogens and play an important role in disease control [38,39]. Ses has a variety of biological activities and has been shown to have a good inhibitory effect on *Candida albicans* and *Mycobacterium tuberculosis* [16,17]. In this paper, the underlying antifungal mechanism of Ses against *P. neglecta* was discussed for the first time.

SEM observation showed that Ses treatment significantly altered the morphology of *P. neglecta* mycelium, causing cell membrane breakage. TEM analysis later found cell membrane wrinkling and cytoplasmic organelle degradation. This suggests that the antifungal mechanism of Ses includes altering the mycelial morphology of *P. neglecta*, causing damage to the cell membrane, and disrupting its integrity and permeability. These results were similar to the damage caused by 2-Phenylethanol to the cell membranes of *Botrytis cinerea* [40].

Lipids and ergosterols in fungal cell membranes are crucial for maintaining membrane integrity, rigidity, fluidity, and overall fungal cell function [41,42]. Li et al. [30] discovered that o-vanillin reduces lipid and ergosterol content in *Aspergillus flavus*, thereby disrupting its cell membrane. In our study, Ses significantly decreased the total lipids and ergosterol content in *P. neglecta*, indicating that Ses disrupts cell membrane structure and integrity, thereby impacting fungal growth. Furthermore, the PI staining test indicated significant fluorescence variances in Ses compared with the control groups, providing evidence that Ses interfered with cell membrane integrity in *P. neglecta*.

The parameters of permeability in cell membranes include the loss of absorbed material at 260 nm, conductivity, and changes in extracellular pH. These parameters were typically considered indicators of severe and irreversible injury to the plasma membrane [20]. In our study, we found that the release of cytosolic components and relative conductivity significantly increased in concentration-dependent Ses-treated groups compared with the control groups. This evidence shows that damage to the cellular membrane structure of *P. neglecta* results in the release of macromolecular cytoplasmic components [43]. The findings closely matched those reported by Mi et al. [44] for the carvacrol treatment of *Clonostachys rosea* and *Fusarium equiseti*. In another study, thymol impaired *Neopestalotiopsis clavispora* cell membrane integrity [45]. In addition, extracellular pH decreased with increasing concentrations of Ses, further supporting the result that cell membrane permeability was disrupted. These results indicate that irreversible damage to the *P. neglecta* cell membrane occurs, which leads to leakage of intracellular material. 

Mitochondria are very important organelles in eukaryotic cells and are the center of cellular metabolism and energy conversion, producing ATP and metabolites (containing TCA cycle metabolites) for survival and growth, respectively [46]. The ATP content significantly impacts the typical energy metabolism of microorganisms. Moreover, the TCA cycle is a crucial pathway for supplying energy for cellular activities, and it is essential for microbial energy metabolism [47]. The reduction in key dehydrogenase activities in the TCA cycle was indicative of impaired energy metabolism [21,37]. In our study, TEM observations revealed some degree of mitochondrial damage; in addition, the reduced ATP content and decrease of key enzyme activities related to the TCA cycle (Figure 7) also indicated that the mitochondria of *P. neglecta* were damaged. Similar results were also seen in the study of sodium pheophorbide, an inhibition of *P. neglecta* [48]. The results of the present study support the view that the cell membrane and mitochondria of *P. neglecta* were important antifungal target sites of Ses.

Small changes in the structural integrity and permeability of cell membranes can have significant effects on cellular metabolism, including the production of extracellular toxins [20]. Research has demonstrated that PG and EG are significant in the pathogenesis of certain fungi and serve as crucial virulence factors for plant pathogens [49,50,51]. Ranjbar et al. [52] conducted a study in which they observed a decrease in cellulase and pectinase (PL) activities secreted by pomegranate rot fungi after treatment with thymol. This ultimately affected the growth of pomegranate rot fungi. In a separate study, Yang et al. [48] found that sodium pheophorbide reduced the activity of EG and PG in *P. neglecta*. In this study, Ses reduced the activities of EG and PG, suggesting that Ses can reduce the virulence of *P. neglecta* and mitigate its pathogenicity. However, in addition to the mechanisms elaborated in this study, the computer modeling of interaction sites could be further investigated to clarify the key action sites.

## 5. Conclusions

Ses can inhibit the mycelial growth of *P. neglecta* and is a potential antifungal compound. Its antifungal activity is mediated by damaging the cell membrane integrity and permeability of *P. neglecta* and interfering with energy metabolism, which ultimately affects mycelial growth and reduces its virulence. These results indicate that Ses can be used as an alternative to conventional fungicides.

## Figures and Tables

**Table 1:** Toxicity of Ses on the mycelial growth of *P. neglecta*.

	Phytopathogenic	EC_50_ (µg/mL)	95% Confidence Interval (µg/mL)	Correlation Coefficient	Toxicity Equation
Ses	*P. neglecta*	142.02 ± 13.22	118.59~170.42	0.927	Y = 1.83x − 4.05

## Data Availability

The original contributions presented in the study are included in the article, further inquiries can be directed to the corresponding author/s.

## Abbreviations

Ses: Sesamol; PDA, potato dextrose agar; PDB, potato dextrose broth; DMSO, dimethyl sulfoxide; SEM, scanning electron microscopy; TEM, transmission electron microscopy; PI, propidium iodide; TCA cycle, tricarboxylic acid cycle; SDH, succinate dehydrogenase; MDH, malic dehydrogenase; CS, citrate synthetase; IDH, isocitrate dehydrogenase; α-KGDH, α-ketoglutarate dehydrogenase; PG, polygalacturonase; EG, endoglucanase.
